# Centered Multi-Task Generative Adversarial Network for Small Object Detection

**DOI:** 10.3390/s21155194

**Published:** 2021-07-31

**Authors:** Hongfeng Wang, Jianzhong Wang, Kemeng Bai, Yong Sun

**Affiliations:** School of Mechatronical Engineering, Beijing Institute of Technology, Beijing 100081, China; 3120185177@bit.edu.cn (H.W.); 3120170114@bit.edu.cn (K.B.); 3120195181@bit.edu.cn (Y.S.)

**Keywords:** generative adversarial network, two-stage small object detection, image super-resolution

## Abstract

Despite the breakthroughs in accuracy and efficiency of object detection using deep neural networks, the performance of small object detection is far from satisfactory. Gaze estimation has developed significantly due to the development of visual sensors. Combining object detection with gaze estimation can significantly improve the performance of small object detection. This paper presents a centered multi-task generative adversarial network (CMTGAN), which combines small object detection and gaze estimation. To achieve this, we propose a generative adversarial network (GAN) capable of image super-resolution and two-stage small object detection. We exploit a generator in CMTGAN for image super-resolution and a discriminator for object detection. We introduce an artificial texture loss into the generator to retain the original feature of small objects. We also use a centered mask in the generator to make the network focus on the central part of images where small objects are more likely to appear in our method. We propose a discriminator with detection loss for two-stage small object detection, which can be adapted to other GANs for object detection. Compared with existing interpolation methods, the super-resolution images generated by CMTGAN are more explicit and contain more information. Experiments show that our method exhibits a better detection performance than mainstream methods.

## 1. Introduction

With visual sensors and computer vision development, gaze estimation technology can obtain gaze points with high accuracy [1]. However, the application of gaze estimation is still limited to visual attention analysis [2], assistive technologies for users with motor disabilities [3], behavior research [4], etc. Meanwhile, object detection algorithms such as YOLOv4 [5] and Faster RCNN [6] have low confidence and apparent location deviation in the prediction of small objects. The method of combining object detection and gaze estimation can significantly improve small object detection performance.

Object detection algorithms have achieved impressive accuracy and efficiency in detecting large objects. However, the performance with small-sized objects is far from satisfactory. There is still a big gap between the performances with small and large objects in recall and accuracy. To achieve a better detection performance when using small objects, SSD [7] uses feature maps from shallow layers for small objects. FPN [8] exploits a feature pyramid to combine feature maps at different scales. Bai et al. [9] introduced a generative adversarial network to implement image super-resolution for small object detection. SOD-MTGAN [10] takes ROIs as input and predicts the categories and locations of objects.

The shallow feature maps are full of textural information but less discriminative, which leads to many false positive results in SSD. The up-sampling of FPN and [9] might generate artifacts which can cover the feature of small objects. SOD-MTGAN takes ROIs from baseline detectors as input, which means that SOD-MTGAN is only executed as the second stage of two-stage object detection. The performance of SOD-MTGAN is heavily dependent on its baseline detector. SOD-MTGAN exploits deconvolution layers for up-sampling, which generates fewer artifacts [10]. However, SOD-MTGAN did not propose a method to suppress artifacts.

In this paper, we proposed a centered multi-task generative adversarial network (CMTGAN) to improve detection performance on small objects, which exploits points of interest presented by gaze estimation methods or detectors (e.g., YOLOv4, etc.) for small object detection. We exploit a gaze estimation method or a detector as a baseline selector to propose points of interest. CMTGAN crops the selected regions centered by points of interest and performs two-stage object detection. Following the previous works on GANs, CMTGAN consists of two subnetworks: a generator and a discriminator. The generator performs super-resolution on selected regions. The discriminator distinguishes real images (high-resolution images) from fake images (super-resolution images) and performs complete two-stage object detection.

**Contributions:** The contributions can be summarized as follows: (1) We proposed an end-to-end convolutional network based on classical GAN for small object detection, which can perform effective single image super-resolution and complete two-stage object detection.

Our method can be pre-trained on high-resolution images for super-resolution without extra object information, which helps the generator learn to extract features from low-resolution images efficiently. The generator performing super-resolution and the discriminator performing object detection can be trained together, which helps them learn to perform better detections simultaneously.

(2) We introduced artificial texture loss into the generator to suppress the artifacts generated by up-sampling, which improves the detection performance on small objects. Artificial texture loss helps the generator reach a balance between textures from original images and textures generated by super-resolution. (3) We exploit a centered mask in the network, making the generator pay more attention to the central part of images. (4) The experiments on VOC datasets reveal that CMTGAN can restore sharper images with more information from low-resolution images than traditional interpolation methods.

Our method has a better performance than mainstream one-stage and two-stage object detection methods. It is also more efficient than object detection methods combined with CNN-based super-resolution methods.

CMTGAN can perform state-of-art detection on small/medium objects.

## 2. Related Work

### 2.1. Small Object Detection

Traditional object detection methods are based on handcrafted features and the deformable part model [11]. Due to the limitation of handcrafted features, traditional methods are far less robust than methods based on deep neural networks. Especially for small object detection, the performance of traditional methods is far from CNN-based methods.

In recent years, object detection methods based on deep neural networks have exhibited superior performances. Currently, CNN-based object detection methods can be categorized as one of two frameworks: the two-stage framework (e.g., Faster RCNN [6], FPN citefpn, etc.) and the one-stage framework (e.g., YOLO [5,12,13], SSD [7], etc.). Faster RCNN [6], a milestone of the two-stage framework, performs object detection with two stages. Faster RCNN proposes ROIs in the first stage, then predicts categories and regresses bounding boxes in the second stage. The one-stage frameworks such as YOLO convert object detection to regression problems, significantly improving detection speed. However, Faster RCNN and YOLOv4 still show unsatisfactory performance on small object detection.

To detect small objects better, SSD uses feature maps from the shallow layer. Although shallow feature maps contain more texture information, they lack semantic information, leading to false positive results in SSD. Compared to SSD-like detectors, our discriminator uses deep, strong semantic features to represent small objects, thus reducing the false positive rate.

FPN exploits the feature pyramid to combine low-resolution, semantically strong features with high-resolution, semantically weak features. With the feature pyramid, FPN exhibits a superior performance over Faster RCNN for small object detection. However, FPN up-samples low-resolution features to fit with high-resolution features, a process that introduces artifacts into the features and consequently degrades the detection performance. SOD-MTGAN [10] uses deconvolution layers for up-sampling, which introduces fewer artifacts into features. However, SOD-MTGAN has not proposed a specific method to suppress artifacts.

Xiang et al. [14] proposed a one-stage space-time video super-resolution framework. It exploits a ConvLSTM method to super-resolve videos, but it is not suitable for single image super-resolution. Su et al. [15] proposed a progressive mixture model for single image super-resolution, which achieved impressive performance on super-resolution.

Compared to FPN and the generator of SOD-MTGAN, our method proposes a method to suppress artificial textures. We exploit deconvolution layers for up-sampling like SOD-GAN and propose artificial texture loss to suppress artifacts, which helps our network balance original textures and super-resolution textures.

Different from [15], we combine single image super-resolution and object detection in a CNN-based framework, which means they can be trained together.

### 2.2. Generative Adversarial Networks

In the primary work, the generative adversarial network generates realistic-looking images from random noise input [16]. GAN exhibits an impressive performance in image super-resolution [17,18], image editing [19,20], image generation, style transfer [21,22], representation learning [23,24], object detection [9,10,25], and so on. GAN includes a generator and a discriminator: the generator generates images, and the discriminator determines the authenticity of images. During training, the generator tries to generate more realistic-looking images, and the discriminator struggles to discover the difference between real images and fake images. After that, the well-trained generator can be used to generate realistic-looking images.

Ledig et al. [17] proposed a generative adversarial network for image super-resolution. The generator takes low-resolution images as input to generate super-resolution images. Real high-resolution images and fake images (e.g., super-resolution images) are delivered to the discriminator. The discriminator the difference between real images and fake images. Bai et al. [10] introduces SOD-MTGAN for image super-resolution and small object detection. The generator of SOD-MTGAN takes ROIs proposed by a baseline detector (e.g., Faster RCNN) as input and performs super-resolution on ROIs. The discriminator of SOD-MTGAN has three tasks: judging the authenticity of the image, predicting categories, and fine-tuning bounding boxes. The discriminator plays a role as the second-stage subnetwork in the two-stage framework. Therefore, the baseline detector has a significant influence on the detection performance of SOD-MTGAN.

Compared to SOD-MTGAN, the discriminator of our method performs complete two-stage small object detection. The generator of CMTGAN takes selected regions as input and performs super-resolution. Then the discriminator proposes ROIs on super-resolution images in the first stage, predicts object categories and regresses object locations in the second stage. The baseline selector only proposes points of interest, which means that CMTGAN has less reliance on the selector.

## 3. Proposed Method

**Figure 1:**
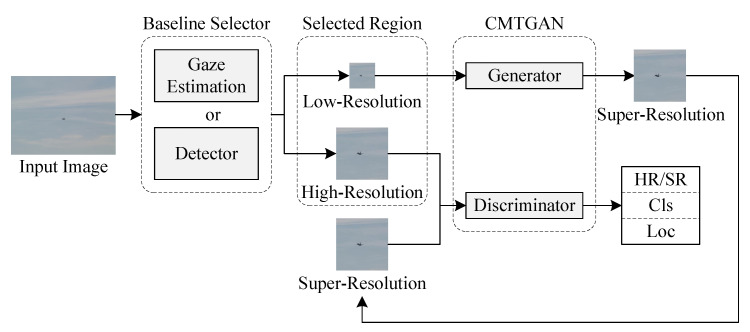
Workflow of CMTGAN.

CMTGAN includes a generator and a discriminator. As shown in Figure 1, the baseline selector creates points of interest on the input containing small objects. We cropped the selected regions centered by points of interest as high-resolution images (HR images) and down-sampled the HR images to obtain low-resolution images (LR images). The generator takes the LR images to generate super-resolution images (SR images). The HR/SR images are delivered to the discriminator. The discriminator categorizes the input as real or fake and detects small objects.

### 3.1. Network Architecture

#### 3.1.1. Generator

**Figure 2:**
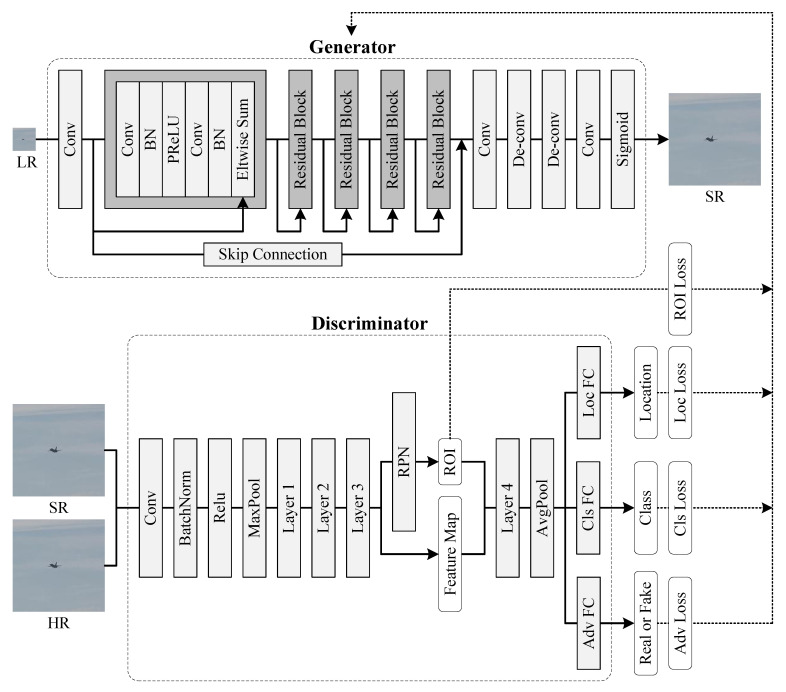
Architecture of CMTGAN.

As shown in Figure 2 and Table 1, we adopted a deep CNN architecture which has shown impressive performance in tiny face detection [9] and super-resolution [17].

There is one skip connection layer, one RPN layer, one sigmoid layer, two deconvolution layers, three convolution layers, and five residual blocks in the generator. Differently from [9], we introduced a skip connection layer into the generator, which brings texture information from shallow layers to up-sampling layers. Differently from the up-sampling layers in [9,17], we exploited deconvolution layers for up-sampling, which achieves a higher efficiency and generates fewer artifacts [10]. Every deconvolution layer performs up-sampling with a factor of 4, which means that the size of SR images is four times that of LR images. We exploit a sigmoid layer to limit the output, which can avoid gradient exploding problems in training.

#### 3.1.2. Discriminator

As shown in Figure 2 and Table 2, we employed ResNet-50 as our backbone network in the discriminator. ResNet-50 is not the only choice, which can be replaced with ResNet-101, AlexNet, or VGGNet for different objects. We introduced an ROI layer into the backbone network to propose ROIs. We used an average pooling layer following the backbone network for down-sampling. We used three parallel fully connected layers behind the average pooling layer, which distinguish the real HR images from the generated SR images, predicting object categories, and regressing bounding boxes.

**Figure 3:**
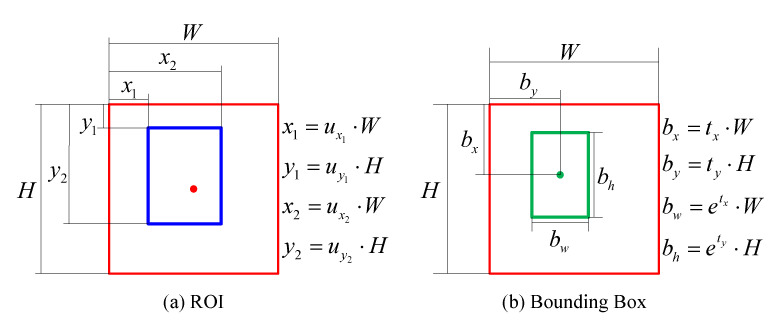
ROI and bounding box. The red point denotes the point of interest proposed by the baseline selector, and the red box indicates the selected region centered by the point of interest. Due to the error of the baseline selector, the point of interest cannot properly coincide with the center of the ground-truth bounding box. The blue box shows the ROI proposed by the discriminator. The green box denotes the predicted bounding box, and the green point represents the center of the predicted bounding box.

The discriminator takes HR images and SR images as input. The backbone network extracts features from input and proposes ROIs. Figure 3a shows the tuple $u=\left(u_{x1},u_{y1},u_{x2},u_{y2}\right)$ of ROI. Behind the average pooling layer, the first fully connected layer ($FC_{Adv}$) uses softmax to predict the probability ($P_{HR}$) of the input image being a real HR image. The second fully connected layer ($FC_{Cls}$) also uses softmax, which outputs the probability $P_{Cls}=\left(p_{0},\ldots ,p_{K}\right)$ of the ROI, each being part of the K+1 object categories. The third fully connected layer ($FC_{Loc}$) outputs the bounding box offset tuple $t=(t_{x},ty,t_{w},t_{h})$. As shown in Figure 3b, the offset tuple $t=(t_{x},ty,t_{w},t_{h})$ corresponds to the bounding box.

Compared to the discriminator in [17,23], our discriminator not only distinguishes real images from fake images but also detects objects in the images. The discriminator in [9] predicts the probability of the input being a face. The discriminator in [10] predicts the probability of the input being each of the categories and fine-tunes the bounding boxes. Compared to [9] and [10], our discriminator performs complete two-stage object detection, proposing ROIs, predicting object categories, and regressing bounding boxes. The difference between our method and [10] means that we only need a point of interest to detect a small object, while [10] needs an ROI proposed by its baseline detector.

### 3.2. Loss Function

We incorporated the loss functions from some state-of-art GAN approaches and propose centered content loss that satisfies the needs of small object detection. Centered content loss consists of pixel-wise loss, perception loss, and artificial texture loss. Centered content loss cooperates with adversarial loss, guiding the generator to generate realistic-looking images easier for small object detection. Furthermore, we propose two-stage detection loss, including ROI loss, classification loss, and regression loss. On the one hand, two-stage detection loss enables the discriminator to perform two-stage object detection. On the other hand, two-stage detection loss drives the generator to recover fine details from LR images for easier detection, as shown in Figure 2. In the following, we describe the centered content loss and the adversarial loss. Furthermore, we define the objective functions of the generator and the discriminator.

#### 3.2.1. Centered Content Loss

**Figure 4:**
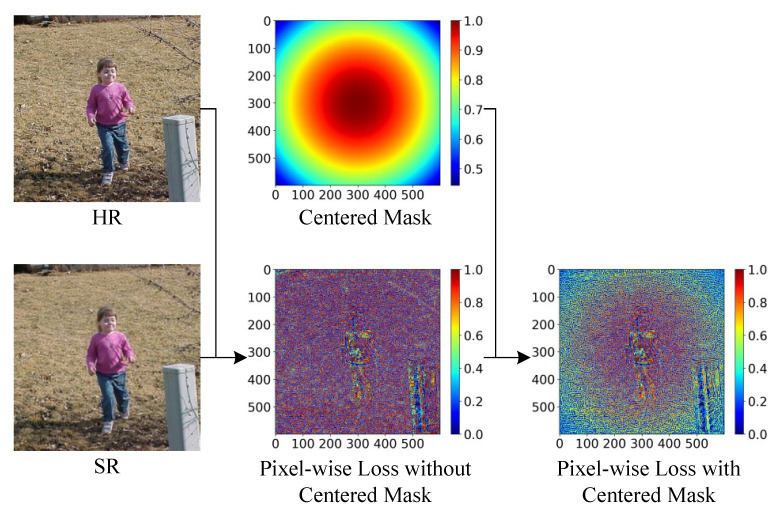
Centered mask.

As shown in Figure 1, the selected regions contain small objects in the central part. We introduced a centered mask which makes the content loss more sensitive to the central part of SR images. The centered mask is shown in Equation (1), and Figure 4 shows the suppression effect of our centered mask.

(1) $$M_{x,y}=\cos\left(\pi \sqrt{{\left(\frac{x}{W}-\frac{1}{2}\right)}^{2}+{\left(\frac{y}{H}-\frac{1}{2}\right)}^{2}}\right)$$

Here, *W* and *H* denote the size of SR images.

**Pixel-wise loss:** Instead of the generator in [16] taking random noise as input, our generator creates SR images from LR images. A natural and straightforward way is to enforce the generator’s output to be the ground-truth images by minimizing the pixel-wise loss, which has been proved effective in some state-of-the-art approaches [26,27]. The pixel-wise loss is computed as Equation (2).

(2) $$l_{pixel-wise}=\frac{1}{WH}\sum_{x=1}^{W}\sum_{y=1}^{H}M_{x,y}\cdot {\left(I_{x,y}^{HR}-G_{\omega }{\left(I^{LR}\right)}_{x,y}\right)}^{2}$$

Here, $M_{x,y}$ denotes the centered mask. $I^{HR}$ and $G_{\omega }\left(I^{LR}\right)$ denote real HR images and generated SR images. *G* represents the generator, and ω denotes its parameters. *W* and *H* denote the size of HR/SR images and the centered mask.

**Perception loss:** Solutions of MSE optimization problems often lack high-frequency content, which results in images covered with overly smooth textures. Therefore, we adopted the perception loss based on the pre-trained ResNet [28]. The pixel-wise loss is computed as Equation (3).

(3) $$l_{perception}=\frac{1}{wh}\sum_{x=1}^{w}\sum_{y=1}^{h}M_{x,y}\cdot {\left(R{\left(I^{HR}\right)}_{x,y}-R{\left(G_{\omega }\left(I^{LR}\right)\right)}_{x,y}\right)}^{2}$$

Here, *R* denotes the pre-trained ResNet. *w* and *h* indicate the size of the feature map created by *R*.

**Artificial texture loss:** The perception loss increases high-frequency content in SR images, making them sharper. However, perception loss without suppression tends to introduce artificial textures into images, which do not exist in HR images. These artificial textures significantly reduce the perception loss, but they also obscure the original textures of images, which is fatal for small object detection. Artificial texture loss is proposed to suppress the artificial textures encouraged by perception loss. The artificial texture loss is computed as Equation (4).

(4) $$\begin{array}{cc} l_{texture}=\; & \frac{1}{W-1}\sum_{x=1}^{W-1}M_{x}\cdot {\left(G_{\omega }{\left(I^{LR}\right)}_{x+1,*}-G_{\omega }{\left(I^{LR}\right)}_{x,*}\right)}^{2}+\\ \; & \frac{1}{H-1}\sum_{y=1}^{H-1}M_{y}\cdot {\left(G_{\omega }{\left(I^{LR}\right)}_{*,y+1}-G_{\omega }{\left(I^{LR}\right)}_{*,y}\right)}^{2} \end{array}$$

in which

(5) $$\begin{array}{c} M_{x}=\cos\left(\pi \left|\frac{x}{W}-\frac{1}{2}\right|\right)\\ M_{y}=\cos\left(\pi \left|\frac{y}{H}-\frac{1}{2}\right|\right) \end{array}$$

where $M_{x}$ and $M_{y}$ are the variants of $M_{x,y}$ to the direction of *x* and *y*. *W* and *H* denote the size of the super-resolution image. $G_{\omega }{\left(I^{LR}\right)}_{x,*}$ is the sum of the pixel values of the *x*-th row in the generated image. $G_{\omega }{\left(I^{LR}\right)}_{*,y}$ denotes the sum of the pixel values of the *y*-th column in the generated image.

#### 3.2.2. Adversarial Loss

We adopted an adversarial loss to generate more realistic-looking SR images, which has been proved to be efficient in [23]. The adversarial loss is defined as Equation (6):

(6) $$l_{adv}=\log\left(D_{\theta }\left(I^{HR}\right)\right)+\log\left(1-D_{\theta }\left(G_{w}\left(I^{LR}\right)\right)\right)$$

where *D* represents the discriminator and θ denotes its parameters. $D_{\theta }\left(I^{HR}\right)$ denotes the probability of the input $I^{HR}$ being a real HR image.

The adversarial loss encourages the discriminator to have a stronger discriminative ability to distinguish real HR images from generated SR images. At the same time, the adversarial loss drives the generator to produce images with fine details.

#### 3.2.3. Detection Loss

As shown in Figure 2, our discriminator is a two-stage object detection method. First, the discriminator proposes ROIs from the input. Second, the discriminator predicts object categories and regresses bounding boxes on ROIs. To achieve this, we propose detection loss, including ROI loss, classification loss, and regression loss.

**ROI Loss:** To complete the task of proposing ROIs and ensuring the generated images are in more detail, we introduced the ROI loss to the overall objective. The ROI loss is defined as Equation (7):

(7) $$l_{ROI}=\sum_{i\in \left(x_{1},y_{1},x_{2},y_{2}\right)}S_{L_{1}}\left(r_{i}-u_{i}\right)$$

in which

(8) $$S_{L_{1}}\left(x\right)=\left\{\begin{array}{cc} 0.5x^{2} & |x|<1\\ |x|-0.5 & |x|<1 \end{array}\right.$$

where $r=\left(r_{x1},r_{y1},r_{x2},r_{y2}\right)$ denotes a tuple of the true ROI regression target, and $u=\left(u_{x1},u_{y1},u_{x2},u_{y2}\right)$ denotes the proposed ROI tuple *u* shown in Figure 3a.

In our method, ROI loss plays two roles. First, it guides the discriminator to propose ROIs from the input, regardless of whether they are real HR images or generated SR images. Second, it promotes the generator to recover images with more detail, making it easier to propose ROIs.

**Classification Loss.** In order to complete the object categorization, we adopted cross-entropy loss as our classification loss. The classification loss is defined as Equation (9):

(9) $$l_{cls}=\sum_{k=1}^{K}-y_{i,k}\log\left(D_{cls}\left(I_{i}^{HR}\right)\right)-y_{i,k}\log\left(D_{cls}\left(G_{\omega }\left(I_{i}^{LR}\right)\right)\right)$$

in which

(10) $$y_{i,k}=\left\{\begin{array}{cc} 1 & \mathrm{if}\;\mathrm{target}\;i\;\mathrm{belongs}\;\mathrm{to}\;\mathrm{class}\;k\\ 0 & \mathrm{otherwise} \end{array}\right.$$

where $D_{cls}\left(I_{i}^{*}\right)$ denotes the probability of the *i*-th input belonging to the *k*-th category.

Our classification loss also plays two roles in the discriminator and the generator, respectively. First, it encourages the discriminator to predict accurate object categories. Second, it drives the generator to produce images that are easier to classify.

**Regression Loss:** We also introduced regression loss into the objective function to complete the two-stage object detection and promote the generated images that make it easier to localize small objects.

(11) $$l_{loc}=\sum_{j\in (x,y,w,h)}S_{L_{1}}\left(t_{i,j}-v_{i,j}\right)$$

where $v=\left(v_{x},v_{y},v_{w},v_{h}\right)$ denotes a tuple of the true bounding box regression target, and $t=\left(t_{x},t_{y},t_{w},t_{h}\right)$ denotes the tuple of the predicted bounding box, as shown in Figure 3b.

Similar to the ROI loss, our regression loss also has two purposes. First, it guides the discriminator to fine-tune the bounding box in the ROI proposed in the first stage. Second, it encourages the generator to produce sharper images with more high-frequency content.

#### 3.2.4. Objective Function

Based on the previous analysis, we propose the objective function of CMTGAN. CMTGAN can be trained by optimizing the objective function. We adopted two objective functions for the generator and the discriminator, respectively. The loss functions $L_{G}$ of the generator and $L_{D}$ of the discriminator are shown in Equations (12) and (13).

(12) $$\begin{array}{cc} L_{G}= & \frac{1}{N}\sum_{i=1}^{N}\lambda_{pix}l_{pixel-wise}+\frac{1}{N}\sum_{i=1}^{N}\lambda_{perc}l_{perception}+\frac{1}{N}\sum_{i=1}^{N}\lambda_{tex}l_{tex}+\\ \; & \frac{1}{N}\sum_{i=1}^{N}\lambda_{adv}l_{adv}+\\ \; & \frac{1}{N}\sum_{i=1}^{N}\lambda_{det}\left(l_{ROI}+l_{cls}+l_{loc}\right) \end{array}$$

(13) $$\begin{array}{cc} L_{D}= & \frac{1}{N}\sum_{i=1}^{N}\tau_{ROI}l_{ROI}+\frac{1}{N}\sum_{i=1}^{N}\tau_{cls}l_{cls}+\frac{1}{N}\sum_{i=1}^{N}\tau_{loc}l_{loc}+\\ \; & \frac{1}{N}\sum_{i=1}^{N}\tau_{adv}l_{adv} \end{array}$$

where $\lambda_{pix}$, $\lambda_{perc}$, $\lambda_{tex}$, $\lambda_{adv}$ and $\lambda_{det}$ denote the trade-off weights during training generator *G*. $\tau_{ROI}$, $\tau_{cls}$, $\tau_{loc}$, and $\tau_{adv}$ denote the trade-off weights during training discriminator *D*. $l_{pixel-wise}$, $l_{perception}$, $l_{tex}$, $l_{adv}$, $l_{ROI}$, $l_{cls}$ and $l_{loc}$ denote the pixel-wise loss in Equation (2), the perception loss in Equation (3), the artificial texture loss in Equation (4), the adversarial loss in Equation (6), the ROI loss in Equation (7), the classification loss in Equation (9) and the regression loss in Equation (11).

The loss function of generator *G* consists of centered content loss, adversarial loss, and detection loss. Different to the previous GAN methods, we introduced the centered mask and artificial texture loss into the centered content loss. The centered mask promotes the generator focus on improving details of the central part, which satisfies the needs of small object detection. Artificial texture loss helps the generator reach a balance between keeping original features and generating super-resolution textures. The loss function of discriminator *D* includes adversarial loss and detection loss. Different from [10], we introduced ROI loss into our detection loss, which helps the discriminator perform the first stage of small object detection: propose ROIs. We also adopt classification loss and regression loss for the second stage: predict object categories and regress bounding boxes.

While training the generator, we froze the discriminator, calculated the loss of the generator with $L_{G}$, and updated the generator by backpropagation. Similar to the generator, we also optimized the discriminator while keeping the generator frozen.

## 4. Experiments

### 4.1. Datasets and Evaluation Metrics

We implemented our model with PyTorch and all the following experiments were performed on a single NVIDIA GeForece RTX 3090 GPU. Table 3 shows our system requirements. Considering the GPU’s performance, we experimentally validated our proposed method on the VOC dataset.

The VOC dataset contains 20 object categories including vehicles, households, animals, and others. This dataset has been widely used as a benchmark for object detection tasks [29].

Due to the resolution of the dataset, we exploited original images for the pre-training of the generator. After that, we created selected regions from original images for the pre-training of the discriminator and the training of CMTGAN, respectively.

**Figure 5:**
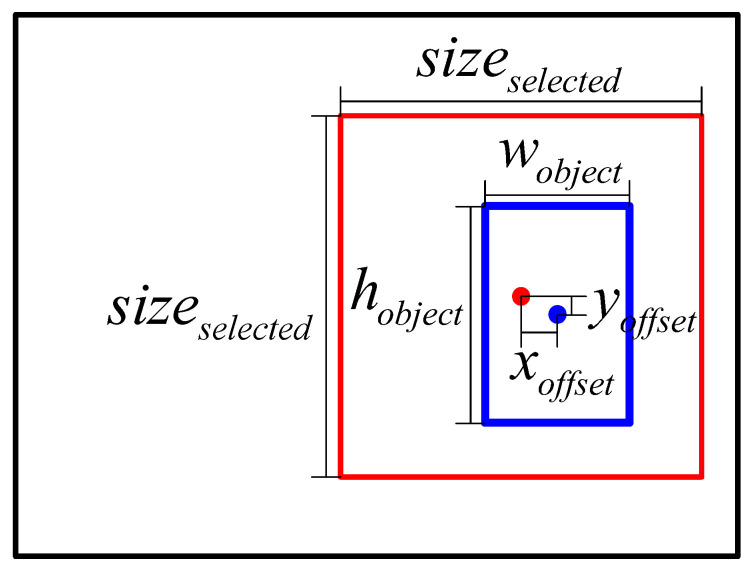
Selected region. The blue point denotes the center of the target, and the blue box indicates the ground-truth bounding box. The red point denotes the point of interest proposed by the baseline selector, and the red box indicates the selected region.

Due to the errors in the baseline selector, the point of interest cannot properly coincide with the center of the target. As shown in Figure 5, we also added a random offset $\left(x_{offset},y_{offset}\right)$ from the center of the target while creating selected regions. $\left(x_{offset},y_{offset}\right)$ is shown in Equation (14).

(14) $$\begin{array}{c} x_{offset}=\mathrm{random}\left(10,\sqrt{w_{object}\cdot h_{object}}\right)\\ y_{offset}=\mathrm{random}\left(10,\sqrt{w_{object}\cdot h_{object}}\right) \end{array}$$

where $w_{object}$ and $h_{object}$ denote the size of the detection target. The function $\mathrm{random}\left(x_{1},x_{2}\right)$ returns a random integer from $x_{1}$ to $x_{2}$. After that, we took the point of interest as the center and crop the selected region with a fixed size $size_{selected}$.

We exploited average gradient (AG), standard deviation (STD), and mutual information (MI) to validate the performance of our generator, in which AG shows the definition of images, STD shows the quantity of information, and MI denotes the similarity between HR/SR images. Furthermore, we performed small object detection with CMTGAN and some mainstream methods with one-stage frameworks or two-stage frameworks. We divided the objects into small (area < $96^{2}$), medium ($96^{2}$ > area > $32^{2}$), and large objects (area > $96^{2}$). We focused on the detection of small/medium objects and report the final detection performance with AP.

### 4.2. Implementation Details

In the generator, we set the trade-off weights $\lambda_{pix}=1$, $\lambda_{perc}=0.006$, $\lambda_{tex}=2\times 10^{-8}$, $\lambda_{adv}=\lambda_{det}=0.001$. In the discriminator, we set the trade-off weights $\tau_{adv}=\tau_{ROI}=\tau_{cls}=\tau_{loc}=1$. First, we performed the pre-training of the generator and the discriminator. Second, we trained the CMTGAN for image super-resolution and small object detection.

**Pre-training of the generator and the ${\mathrm{FC}}_{\mathrm{adv}}$ branch of the discriminator.** We created HR images in the size of $400^{2}$ from the VOC dataset and exploited down-sampling to produce LR images at the size of $100^{2}$. Then, we performed the pre-training on HR and SR images. The generator produces SR images at the size of $400^{2}$ from LR images, and the $FC_{adv}$ branch outputs the probability of the input being a real HR image. Our generator was trained from scratch. The weights in each layer were initialized with a zero-mean Gaussian distribution with standard deviation 0.02, while the biases were initialized with 0. The backbone network of discriminator loaded the pre-trained weights of ResNet-50. The weights in the fully connected layer of $FC_{adv}$ branch were initialized with a zero-mean Gaussian distribution with a standard deviation of 0.1, while the biases were initialized with 0. During the pre-training, the weights and biases in the backbone network of the discriminator were fixed, which makes the discriminator more stable. We adopted the Adam optimizers for the generator and the discriminator, respectively. The learning rates for the optimizers were initially set to 0.0001 and were then reduced to 95% after every epoch. We alternately updated the generator and the discriminator networks: we updated the generator every five iterations and updated the discriminator every iteration except on the generator’s turn. The pre-training was terminated after 50 epochs, and the states of the network were recorded.

**Pre-training of the discriminator:** We pre-trained the $FC_{cls}$ branch and $FC_{loc}$ branch of the discriminator on the selected regions with $size_{selected}=150$. Similar to the former pre-training, we also fixed the backbone network of the discriminator. The backbone network of discriminator loads the pre-trained weights of ResNet-50. The weights in RPN layers, fully connected layers of $FC_{cls}$ branch and $FC_{loc}$ branch are initialized with a zero-mean Gaussian distribution with a standard deviation of 0.1, while the biases are initialized with 0. We adopted the Adam optimizer for the discriminator. The learning rate for the optimizer was initially set to 0.0001 and then reduced to 95% after every epoch. The pre-training was terminated after 50 epochs, and the states of the network were recorded.

**Training for CMTGAN:** Finally, we trained CMTGAN on the selected regions. The generator performed super-resolution on the selected regions in the size of $150^{2}$. The discriminator performed object detection on the SR images in the size of $600^{2}$, predicting object categories and regressing bounding boxes. The generator and discriminator load weights from the pre-trained weights. We adopted the Adam optimizers for the generator and the discriminator, respectively. The learning rates for the optimizers were initially set to $1\times 10^{-5}$ and then reduced to 95% after every epoch. We alternately updated the generator and the discriminator networks: we updated the generator every five iterations and updated the discriminator every iteration except on the generator’s turn. The training contains 100 epochs. In the first 50 epochs, layers in the backbone network of the discriminator were fixed. In the following 50 epochs, no layer was fixed.

### 4.3. Experimental Results

#### 4.3.1. Performance of Super-Resolution

**Figure 6:**
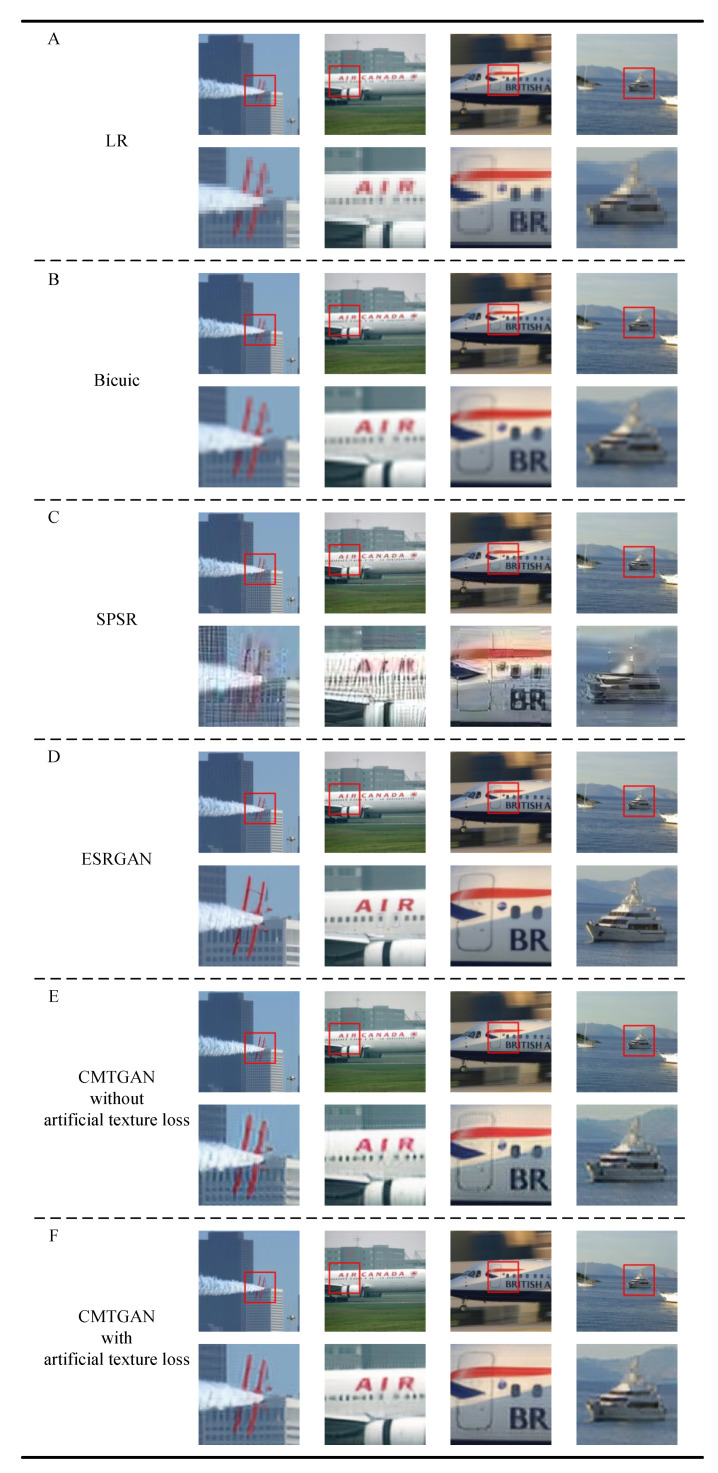
Performance of traditional interpolation methods and CNN-based methods.

The generator performed super-resolution on LR images, and the performance is shown in Figure 6. We performed up-sampling with bicubic interpolation on LR images in the size of $100^{2}$ (Figure 6 row A) and restore images in $400^{2}$ (Figure 6, row B).

We super-resolved LR images with SPSR [30] and ESRGAN (Figure 6, row C and row D).

At the same time, we exploit CMTGAN without artificial texture loss to generate SR images with a factor of 4 (Figure 6, row E). Furthermore, we exploit CMTGAN with artificial texture loss to generate SR images with a factor of 4 (Figure 6, row F).

It is evident that SR images in row E are significantly sharper than restored images in row B. However, SR images in row E contain some abnormal textures, which may cover the original texture information of small objects. Especially in the first image of row E, we can see that the wings are abnormally distorted by artificial textures. SR images in row F contain significantly fewer artificial textures than SR images in row E. The wings in the first image of row F are more realistic than row E.

Although SPSR exhibited an impressive performance on images of buildings, images generated by SPSR in row C contain too many artificial textures for small object detection compared to images generated by our method in row E. ESRGAN generated more realistic-looking images in row D compared to SPSR. Images generated by ESRGAN in row D look sharper than images in row E, which shows extremely clear boundaries. However, due to the optical factors, real HR images captured by cameras do not contain such extremely clear boundaries, which means interference in object detection methods. More details are shown in the following experiments.

In summary, the generator of CMTGAN can generate sharper SR images than traditional interpolation methods. There is no significant gap between the generator of CMTGAN- and CNN-based methods (e.g., ESRGAN, etc.) in single image super-resolution. Artificial texture loss shows significant suppression of artifacts, which helps the generator keep a balance between original features and super-resolution textures.

**Figure 7:**
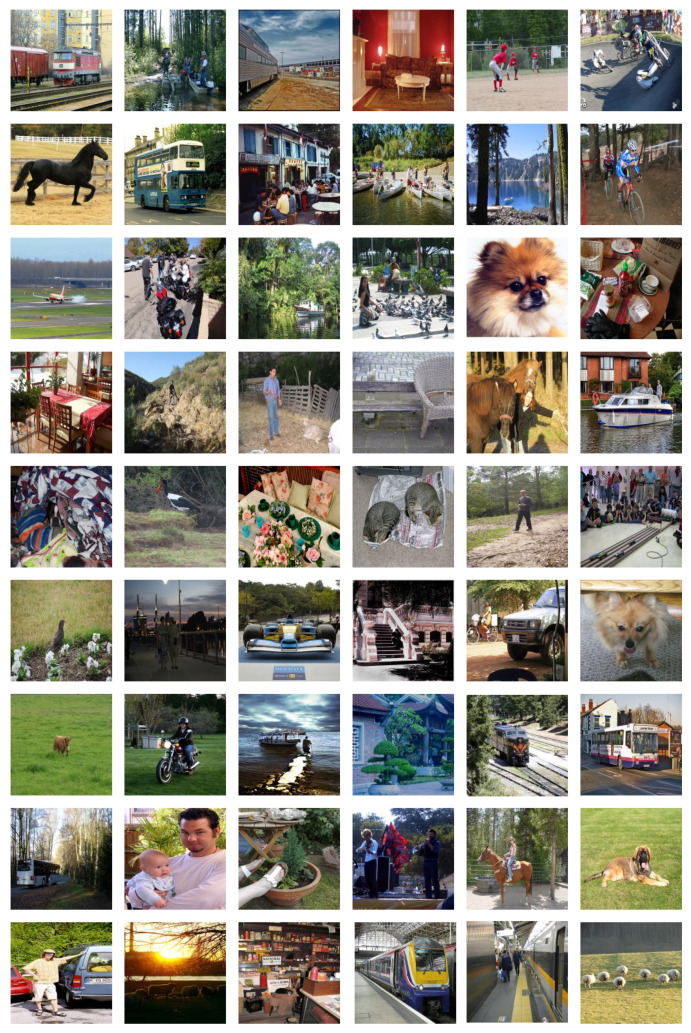
LR images from VOC.

Furthermore, we quantitatively analyzed the super-resolution performance of CMTGAN with AG, STD, MI, and inference time. A higher AG means sharper images, and a higher STD means more information in images. MI shows a similarity between HR images and SR/RE images. We collected 54 HR images from the VOC dataset randomly and down-sampled them to the size of $150^{2}$, as shown in Figure 7. We up-sampled LR images with bilinear interpolation and bicubic interpolation to restore images in the size of $600^{2}$. The generator of CMTGAN produces SR images with a factor of 4. As shown in Table 4, we calculated AG, STD, and MI of SR/RE images to validate the performance of CMTGAN. Taking into consideration the needs of object detection on inference time, we also recorded the inference time in Table 5.

According to Table 4, it is clear that SR images generated by CNN-based methods have higher AG and STD than RE images generated by traditional interpolation methods, and images generated by ESRGAN have the best AG and STD. However, a higher AG and STD do not mean absolutely better images. The images generated by SPSR have a better AG and STD than CMTGAN, while they contain too many artificial textures, as shown in Figure 6. These artificial textures increase AG and STD, but also make small objects hard to detected. Therefore, we exploited MI to measure the similarity between HR images and SR/RE images. As shown in Table 4, SR images generated by CMTGAN have the best MI, which means that SR images generated by CMTGAN are the most similar to the original HR images.

According to Table 5, CMTGAN has the shortest inference time among CNN-based super-resolution methods. The generator of CMTGAN takes an average of 10.1 ms to perform super-resolution, which satisfies the needs of object detection. Although it takes more time than traditional interpolation methods, the inference time of CMTGAN is significantly shorter than SPSR and ESRGAN.

In summary, SR images generated by CMTGAN are sharper than images produced by traditional interpolation methods and contain more information. The generator of CMTGAN exhibits a similar super-resolution performance to some state-of-the-art CNN-based methods. SR images generated by CMTGAN are the most similar to the original HR images as compared to images generated by traditional interpolation methods and CNN-based methods. The generator of CMTGAN can perform real-time super-resolution on a single NVIDIA RTX3090, which satisfies the needs of small object detection.

#### 4.3.2. Performance of Small Object Detection

**Figure 8:**
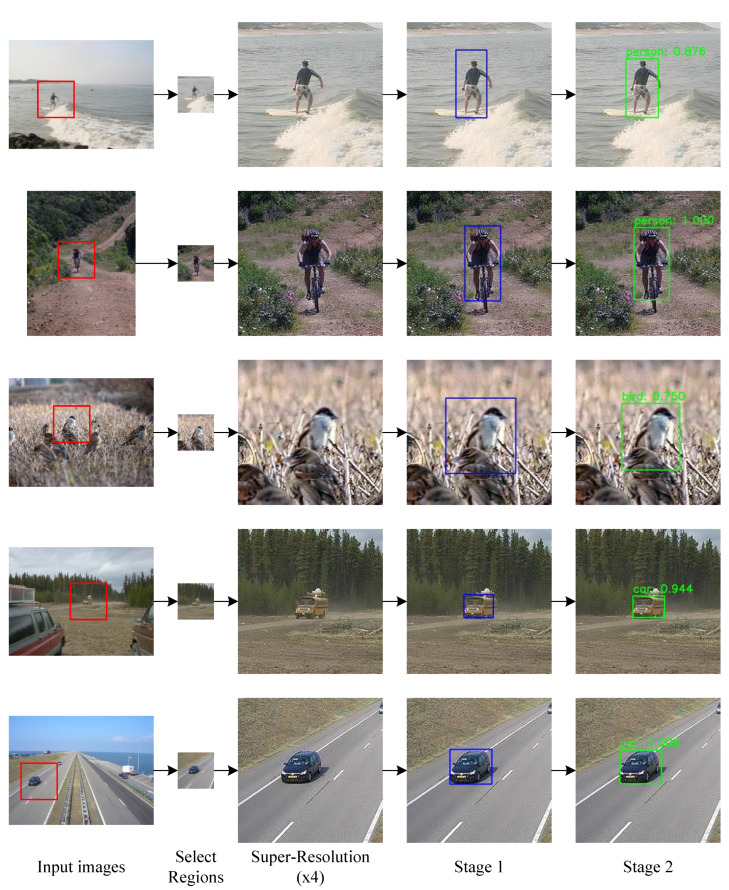
Two-stage object detection of CMTGAN.

We exploited CMTGAN to detect small objects, as shown in Figure 8. The generator performed super-resolution on the input, which made the images easier for detection. The discriminator proposed ROIs in the first stage, predicted object categories and regressed bounding boxes in the second stage.

We performed small/medium object detection on selected regions with CMTGAN, YOLOv4, and Faster RCNN combined with different up-sampling methods. We up-sampled the selected regions from $150^{2}$ to $608^{2}$ with bilinear interpolation and bicubic interpolation, from $150^{2}$ to $600^{2}$ with SPSR and ESRGAN for YOLOv4, which is similar to the super-resolution performed by the generator in CMTGAN. We up-sampled the selected regions from $150^{2}$ to $600^{2}$ with bilinear interpolation, bicubic interpolation, SPSR, and ESRGAN for Faster RCNN, similar to the super-resolution in CMTGAN. Then, we exploited these methods for object detection.

As shown in Table 6, CMTGAN has a better performance on small/medium object detection than YOLOv4 (i.e., 20.52% in AP) and Faster RCNN (i.e., 5.27% in AP).

Although YOLOv4 combined with ESRGAN achieved a higher AP, its inference time also increased as shown in Table 7.

According to Table 7, YOLOv4 combined with bilinear interpolation has the shortest inference time. The inference time of CMTGAN is longer than YOLOv4 combined with bilinear interpolation but significantly shorter than YOLOv4 and Faster RCNN combined with CNN-based super-resolution methods. CNN-based super-resolution methods (e.g., ESRGAN, SPSR, etc.) may benefit small object detection, but they also take a long time to super-resolve LR images, which makes the detection is not in real timeCMTGAN exhibited a better object detection performance than Faster RCNN combined with traditional interpolation with a similar inference time.

## 5. Conclusions

In this paper, we proposed CMTGAN, a new small object detection method based on generative adversarial networks. We introduced artificial texture loss and a centered mask into the generator, with which the generator could create super-resolution images easier for small object detection. The artificial texture loss helped the generator to balance the original features and super-resolution textures. The discriminator of our method performed complete two-stage object detection and distinguished real images from fake images, which can be adapted to other GANs for detection tasks. The experimental results showed that, compared with the existing methods, the generator of CMTGAN could generate sharper super-resolution images with more information. CMTGAN had an obvious advantage in small/medium object detection.

In future work, we will focus on eliminating the baseline selector. Although CMTGAN has a similar inference time than Faster RCNN, there is still a significant difference between YOLOv4 and CMTGAN in inference time. We will investigate how to optimize the architecture of CMTGAN to perform more efficient object detection. Furthermore, we will further investigate the generation of artifacts to achieve a better performance.

## Figures and Tables

**Table 1:** Architecture of the generator in CMTGAN.

Layer	Conv	Res-Block x5	Conv	De-Conv	De-Conv	Conv	Skip
Kernel Num.	64	64	64	256	256	3	64
Kernel Size	9	3	3	3	3	9	1
Stride	1	1	1	2	2	1	1

**Table 2:** Architecture of the discriminator in CMTGAN. K denotes the number of object categories.

Layer	Conv	Max-Pool	Layer 1	Layer 2	Layer 3	RPN	Layer 4	Avg-Pool	FC 1	FC 2	FC 3
Kernel Num.	64	-	128	256	512	512	1024	-	2	K + 1	4(K + 1)
Kernel Size	7	3	1	1	1	3	1	7	-	-	-
Stride	2	2	1	2	2	1	2	1	-	-	-

**Table 3:** System requirements.

CPU	Intel 10700K
GPU	NVIDIA RTX3090
OS	Ubuntu20.04
Language	Python3.8 with PyTorch1.8.1(LTS)

**Table 4:** Metrics of super-resolution.

	**AG**					**STD**					**MI**				
	**Bilinear**	**Bicubic**	**SPSR**	**ESRGAN**	**CMTGAN**	**Bilinear**	**Bicubic**	**SPSR**	**ESRGAN**	**CMTGAN**	**Bilinear**	**Bicubic**	**SPSR**	**ESRGAN**	**CMTGAN**
1	6.696	4.113	10.284	14.265	6.965	49.768	48.022	52.319	53.778	50.661	0.982	0.988	0.883	0.948	1.124
2	4.201	2.773	5.787	6.778	4.102	56.516	55.607	57.123	57.276	56.865	1.647	1.669	1.499	1.747	1.849
3	3.380	2.189	5.416	10.784	4.282	41.626	40.873	42.956	43.643	42.310	1.159	1.158	1.037	1.058	1.258
4	4.584	3.236	9.746	9.708	5.455	63.957	62.528	66.067	66.632	65.631	1.639	1.614	1.314	1.648	1.708
5	2.241	1.401	2.860	5.665	2.978	14.823	14.193	15.104	16.174	15.297	0.702	0.698	0.556	0.611	0.759
6	5.872	4.100	8.804	8.299	6.382	74.184	72.582	76.185	76.263	75.401	1.303	1.291	1.166	1.414	1.401
7	5.976	4.093	9.215	7.331	6.068	70.641	69.057	72.028	72.315	71.848	1.616	1.604	1.412	1.878	1.792
8	3.292	2.124	5.069	8.269	4.114	61.261	60.671	61.797	62.268	61.930	1.274	1.262	1.148	1.232	1.359
9	6.471	4.267	10.566	9.203	6.735	72.393	70.745	74.777	74.401	73.880	1.373	1.367	1.172	1.496	1.529
10	4.496	2.858	7.246	6.138	4.557	53.730	52.782	54.923	54.516	54.242	1.534	1.548	1.320	1.714	1.736
11	4.240	2.718	6.478	5.515	4.515	41.485	40.480	42.597	42.405	41.555	1.315	1.320	1.146	1.467	1.468
12	4.137	2.760	6.202	5.633	4.548	51.775	50.633	53.059	53.031	52.056	1.552	1.528	1.294	1.695	1.599
13	3.396	2.262	5.517	4.503	3.707	53.519	52.704	54.448	54.380	54.089	1.873	1.865	1.649	2.034	2.021
14	4.594	2.797	6.060	10.217	5.133	46.618	45.647	47.448	48.525	47.432	1.246	1.241	1.112	1.204	1.381
15	4.966	3.303	9.192	9.914	5.387	69.867	68.715	71.478	71.716	70.771	1.551	1.572	1.380	1.600	1.707
16	2.140	1.455	4.849	4.131	2.785	40.713	40.169	41.627	41.319	41.505	1.641	1.640	1.429	1.637	1.645
17	3.854	2.354	4.864	12.449	4.305	28.532	27.527	29.299	31.445	29.446	0.908	0.916	0.790	0.748	1.036
18	4.790	2.972	6.984	10.846	5.242	42.267	41.035	43.844	45.142	43.197	1.069	1.063	0.934	1.071	1.196
19	4.470	2.773	5.917	9.165	4.645	58.916	58.032	59.796	60.313	59.259	1.615	1.634	1.443	1.600	1.673
20	4.609	2.991	7.067	8.157	5.028	61.378	60.246	62.825	63.154	62.045	1.750	1.758	1.487	1.773	1.784
21	4.139	2.595	6.493	6.961	4.267	48.795	47.758	49.966	50.349	49.925	1.688	1.713	1.459	1.873	1.857
22	5.248	3.427	8.306	8.366	5.781	52.863	51.164	55.052	55.017	53.862	1.319	1.312	1.159	1.432	1.483
23	5.359	3.692	8.543	7.555	5.832	58.952	57.475	60.863	60.871	60.000	1.464	1.455	1.288	1.698	1.657
24	4.099	2.687	6.552	7.056	4.599	85.025	84.121	85.593	86.896	85.293	1.912	1.881	1.638	1.972	1.882
25	5.972	3.717	8.957	16.066	6.387	52.312	50.775	54.267	56.039	53.228	1.126	1.126	1.012	1.032	1.305
26	1.821	1.270	3.040	1.894	2.240	51.963	51.588	52.661	52.138	51.719	2.464	2.510	2.060	2.684	2.363
27	3.457	2.282	5.615	8.670	3.901	33.958	32.910	35.449	36.090	34.378	1.304	1.331	1.147	1.222	1.423
28	4.726	2.938	6.800	8.703	5.226	44.176	43.029	45.624	45.997	44.996	1.128	1.129	1.034	1.232	1.286
29	4.127	2.679	6.602	5.835	4.351	53.838	52.807	55.218	55.231	54.504	1.671	1.658	1.423	1.867	1.821
30	5.229	3.583	9.719	8.414	6.049	60.858	59.539	63.098	63.069	62.090	1.384	1.385	1.257	1.582	1.585
31	2.909	1.911	3.636	4.588	2.751	71.213	70.810	71.348	71.579	72.170	2.107	2.138	1.954	2.159	2.221
32	4.204	2.850	6.254	5.740	4.616	72.150	71.122	73.294	72.845	72.954	1.690	1.665	1.485	1.806	1.803
33	4.570	3.142	8.576	9.801	5.265	60.982	59.737	63.412	63.102	61.863	1.415	1.410	1.218	1.423	1.510
34	4.086	2.539	5.961	10.784	4.695	40.812	39.807	41.836	43.031	41.475	1.090	1.093	0.979	0.998	1.162
35	3.884	2.515	6.005	5.781	4.393	37.029	35.687	38.856	38.483	37.723	1.321	1.314	1.137	1.399	1.472
36	5.920	4.105	9.760	7.342	6.170	62.528	60.724	64.934	64.394	63.470	1.372	1.374	1.192	1.618	1.584
37	5.315	3.668	10.310	10.576	5.986	69.416	68.063	71.542	71.778	70.556	1.505	1.502	1.325	1.586	1.691
38	3.507	2.153	4.295	4.532	3.963	39.684	39.027	40.410	40.009	39.769	1.470	1.475	1.295	1.645	1.667
39	6.105	4.066	10.277	9.581	6.462	71.704	70.143	73.620	74.197	73.075	1.498	1.502	1.347	1.675	1.689
40	4.922	3.235	8.136	8.628	5.312	69.053	68.017	70.443	70.690	69.939	1.626	1.629	1.422	1.702	1.729
41	4.504	2.741	5.877	7.137	4.894	40.144	38.972	41.447	41.803	40.756	1.167	1.178	1.023	1.274	1.362
42	2.455	1.685	4.341	4.294	2.814	46.511	45.960	47.201	46.959	46.771	1.818	1.814	1.548	1.859	1.900
43	5.888	4.024	10.207	13.441	6.479	57.403	55.648	60.020	60.746	58.590	1.346	1.359	1.170	1.361	1.541
44	7.117	4.549	11.958	13.347	7.295	68.859	67.121	71.403	71.491	70.155	1.210	1.217	1.085	1.267	1.375
45	5.799	3.798	8.163	6.613	5.528	61.637	60.158	62.743	62.588	62.438	1.397	1.419	1.237	1.607	1.610
46	6.743	4.456	9.708	7.015	6.439	62.944	61.046	64.226	64.678	63.716	1.469	1.491	1.301	1.833	1.778
47	3.093	1.968	4.022	3.840	3.314	46.635	46.151	47.020	47.040	47.051	1.795	1.831	1.625	1.933	1.972
48	3.827	2.442	5.417	9.030	4.429	52.819	52.083	53.747	53.999	53.055	1.358	1.349	1.220	1.325	1.485
49	5.648	3.692	11.680	18.414	6.711	58.624	56.957	61.948	64.662	60.159	1.234	1.220	1.083	1.176	1.335
50	5.906	3.845	8.503	9.928	6.107	65.096	63.548	66.613	66.973	66.147	1.416	1.410	1.253	1.504	1.581
51	6.454	4.227	11.331	14.627	7.173	59.019	57.101	61.990	63.246	60.322	1.040	1.036	0.910	1.011	1.184
52	5.613	3.671	9.798	6.785	5.648	62.006	60.582	64.070	63.021	63.163	1.521	1.527	1.311	1.777	1.784
53	4.422	2.867	6.502	6.423	4.930	38.005	36.352	40.019	39.999	39.053	1.179	1.166	1.019	1.337	1.348
54	4.984	3.171	7.207	10.234	5.561	75.578	74.697	76.843	77.739	76.383	1.459	1.453	1.336	1.498	1.594
Avg	4.638	3.032	7.346	**8.425**	5.046	55.307	54.128	56.787	**57.138**	56.114	1.439	1.441	1.262	1.517	**1.575**

**Table 5:** Inference time of super-resolution.

Methods	Bilinear	Bicubic	SPSR	ESRGAN	SR in CMTGAN
Inference time	1.8 ms	2.6 ms	147.1 ms	58.5 ms	10.1 ms

**Table 6:** Performance of object detection.

Methods	AP	APs	APm
Bilinear + YOLOv4	33.39	20.64	35.68
Bicubic + YOLOv4	32.20	22.14	34.30
SPSR + YOLOv4	19.75	13.75	20.84
ESRGAN + YOLOv4	34.70	20.42	36.33
Bilinear + FasterRCNN	49.95	25.20	56.49
Bicubic + FasterRCNN	48.81	24.00	54.86
SPSR + FasterRCNN	26.99	15.91	28.70
ESRGAN + FasterRCNN	46.59	33.58	48.89
CMTGAN	**55.22**	**36.99**	**69.72**

**Table 7:** Inference time of object detection.

Methods	Resize/SR	Detection	Total
Bilinear + YOLOv4	1.8 ms	29.4 ms	31.2 ms
Bicubic + YOLOv4	2.6 ms	29.4 ms	32.0 ms
SPSR + YOLOv4	147.1 ms	29.4 ms	176.5 ms
ESRGAN + YOLOv4	58.5 ms	29.4 ms	87.9 ms
Bilinear + FasterRCNN	1.8 ms	42.1 ms	43.9 ms
Bicubic + FasterRCNN	2.6 ms	42.1 ms	44.7 ms
SPSR + FasterRCNN	147.1 ms	42.1 ms	189.2 ms
ESRGAN + FasterRCNN	58.5 ms	42.1 ms	100.6 ms
CMTGAN	10.1 ms	35.8 ms	45.9 ms

## Data Availability

Not applicable.

## Abbreviations

The following abbreviations are used in this manuscript:

GAN	Generative adversarial network
HR images	High-resolution images
LR images	Low-resolution images
SR images	Super-resolution images
RE images	Restored images
AG	Aaverage gradient
STD	Standard deviation
MI	Mutual information

## References

[1] Fischer T., Chang H.J., Demiris Y. Rt-gene: Real-time eye gaze estimation in natural environments. Proceedings of the European Conference on Computer Vision (ECCV).

[2] Jaques N., Conati C., Harley J.M., Azevedo R. (2014). Predicting affect from gaze data during interaction with an intelligent tutoring system. International Conference on Intelligent Tutoring Systems.

[3] Eid M.A., Giakoumidis N., El Saddik A. (2016). A novel eye-gaze-controlled wheelchair system for navigating unknown environments: Case study with a person with ALS. IEEE Access.

[4] Georgiou T., Demiris Y. (2017). Adaptive user modelling in car racing games using behavioural and physiological data. User Model. User Adapt. Interact..

[5] Bochkovskiy A., Wang C.Y., Liao H.Y.M. (2020). Yolov4: Optimal speed and accuracy of object detection. arXiv.

[6] Ren S., He K., Girshick R., Sun J. (2015). Faster r-cnn: Towards real-time object detection with region proposal networks. arXiv.

[7] Liu W., Anguelov D., Erhan D., Szegedy C., Reed S., Fu C.Y., Berg A.C. (2016). Ssd: Single shot multibox detector. European Conference on Computer Vision.

[8] Lin T.Y., Dollár P., Girshick R., He K., Hariharan B., Belongie S. Feature pyramid networks for object detection. Proceedings of the IEEE Conference on Computer Vision and Pattern Recognition.

[9] Bai Y., Zhang Y., Ding M., Ghanem B. Finding tiny faces in the wild with generative adversarial network. Proceedings of the European Conference on Computer Vision (ECCV).

[10] Bai Y., Zhang Y., Ding M., Ghanem B. Sod-mtgan: Small object detection via multi-task generative adversarial network. Proceedings of the European Conference on Computer Vision (ECCV).

[11] Zhang X., He Z., Ma Z., Yang Y. (2021). A Self-Labeling Feature Matching Algorithm for Instance Recognition on Multi-Sensor Images. Trans. Beijing Inst. Technol..

[12] Redmon J., Farhadi A. (2018). Yolov3: An incremental improvement. arXiv.

[13] Liu H., Fan K., Ouyang Q., Li N. (2021). Real-Time Small Drones Detection Based on Pruned YOLOv4. Sensors.

[14] Xiang X., Tian Y., Zhang Y., Fu Y., Allebach J.P., Xu C. Zooming slow-mo: Fast and accurate one-stage space-time video super-resolution. Proceedings of the IEEE/CVF Conference on Computer Vision and Pattern Recognition.

[15] Su R., Zhong B., Ji J., Ma K.K. Single Image Super-Resolution Via A Progressive Mixture Model. Proceedings of the 2020 IEEE International Conference on Image Processing (ICIP).

[16] Creswell A., White T., Dumoulin V., Arulkumaran K., Sengupta B., Bharath A.A. (2018). Generative adversarial networks: An overview. IEEE Signal Process. Mag..

[17] Ledig C., Theis L., Huszár F., Caballero J., Cunningham A., Acosta A., Aitken A., Tejani A., Totz J., Wang Z. Photo-realistic single image super-resolution using a generative adversarial network. Proceedings of the IEEE Conference on Computer Vision and Pattern Recognition.

[18] Wang X., Yu K., Wu S., Gu J., Liu Y., Dong C., Qiao Y., Change Loy C. ESRGAN: Enhanced Super-Resolution Generative Adversarial Networks. Proceedings of the European Conference on Computer Vision (ECCV).

[19] Feng H., Guo J., Xu H., Ge S.S. (2021). SharpGAN: Dynamic Scene Deblurring Method for Smart Ship Based on Receptive Field Block and Generative Adversarial Networks. Sensors.

[20] Marnerides D., Bashford-Rogers T., Debattista K. (2021). Deep HDR Hallucination for Inverse Tone Mapping. Sensors.

[21] Isola P., Zhu J.Y., Zhou T., Efros A.A. Image-to-image translation with conditional adversarial networks. Proceedings of the IEEE Conference on Computer Vision and Pattern Recognition.

[22] Zhu J.Y., Park T., Isola P., Efros A.A. Unpaired image-to-image translation using cycle-consistent adversarial networks. Proceedings of the IEEE Conference on Computer Vision and Pattern Recognition.

[23] Li J., Liang X., Wei Y., Xu T., Feng J., Yan S. Perceptual generative adversarial networks for small object detection. Proceedings of the IEEE Conference on Computer Vision and Pattern Recognition.

[24] Pan L., Li X., Luo S., Wu Z. (2021). Double-Channel GAN with Multi-Level Semantic Correlation for Event Detection. Trans. Beijing Inst. Technol..

[25] Truong N.Q., Lee Y.W., Owais M., Nguyen D.T., Batchuluun G., Pham T.D., Park K.R. (2020). SlimDeblurGAN-based motion deblurring and marker detection for autonomous drone landing. Sensors.

[26] Dong C., Loy C.C., He K., Tang X. (2015). Image super-resolution using deep convolutional networks. IEEE Trans. Pattern Anal. Mach. Intell..

[27] Shi W., Caballero J., Huszár F., Totz J., Aitken A.P., Bishop R., Rueckert D., Wang Z. Real-time single image and video super-resolution using an efficient sub-pixel convolutional neural network. Proceedings of the IEEE Conference on Computer Vision and Pattern Recognition.

[28] He K., Zhang X., Ren S., Sun J. Deep residual learning for image recognition. Proceedings of the IEEE Conference on Computer Vision and Pattern Recognition.

[29] Dong Z., Xu K., Yang Y., Bao H., Xu W., Lau R.W. (2020). Location-aware Single Image Reflection Removal. arXiv.

[30] Ma C., Rao Y., Cheng Y., Chen C., Lu J., Zhou J. Structure-preserving super resolution with gradient guidance. Proceedings of the IEEE/CVF Conference on Computer Vision and Pattern Recognition.

